# Increased Elastin Degradation in Pseudoxanthoma Elasticum Is Associated with Peripheral Arterial Disease Independent of Calcification

**DOI:** 10.3390/jcm9092771

**Published:** 2020-08-26

**Authors:** Jonas W. Bartstra, Wilko Spiering, Jody M. W. van den Ouweland, Willem P. T. M. Mali, Rob Janssen, Pim A. de Jong

**Affiliations:** 1 Department of Radiology, University Medical Center Utrecht, Utrecht University, 3508 GA Utrecht, The Netherlands; j.w.bartstra@umcutrecht.nl (J.W.B.); w.mali@umcutrecht.nl (W.P.T.M.M.); 2 Department of Vascular Medicine, University Medical Center Utrecht, Utrecht University, 3508 GA Utrecht, The Netherlands; w.spiering@umcutrecht.nl; 3 Clinical Chemistry, Canisius-Wilhelmina Hospital, 6532 SZ Nijmegen, The Netherlands; j.v.d.ouweland@cwz.nl; 4 Department of Pulmonary Medicine, Canisius-Wilhelmina Hospital, 6532 SZ Nijmegen, The Netherlands; rob.janssen@cwz.nl

**Keywords:** pseudoxanthoma elasticum, plasma desmosines, arterial calcification, peripheral arterial disease

## Abstract

Pseudoxanthoma elasticum (PXE) results in extensive fragmentation and calcification of elastin fibers in the peripheral arteries, which results in peripheral arterial disease (PAD). Current research focuses on the role of calcifications in the pathogenesis of PXE. Elastin degradation and calcification are shown to interact and may amplify each other. This study aims to compare plasma desmosines, a measure of elastin degradation, between PXE patients and controls and to investigate the association between desmosines and (1) arterial calcification, (2) PAD, and (3) PAD independent of arterial calcification in PXE. Plasma desmosines were quantified with liquid chromatography-tandem mass spectrometry in 93 PXE patients and 72 controls. In PXE patients, arterial calcification mass was quantified on CT scans. The ankle brachial index (ABI) after treadmill test was used to analyze PAD, defined as ABI < 0.9, and the Fontaine classification was used to distinguish symptomatic and asymptomatic PAD. Regression models were built to test the association between desmosines and arterial calcification and arterial functioning in PXE. PXE patients had higher desmosines than controls (350 (290–410) ng/L vs. 320 (280–360) ng/L, *p* = 0.02). After adjustment for age, sex, body mass index, smoking, type 2 diabetes mellitus, and pulmonary abnormalities, desmosines were associated with worse ABI (β (95%CI): −68 (−132; −3) ng/L), more PAD (β (95%CI): 40 (7; 73) ng/L), and higher Fontaine classification (β (95%CI): 30 (6; 53) ng/L), but not with arterial calcification mass. Lower ABI was associated with higher desmosines, independent from arterial calcification mass (β (95%CI): −0.71(−1.39; −0.01)). Elastin degradation is accelerated in PXE patients compared to controls. The association between desmosines and ABI emphasizes the role of elastin degradation in PAD in PXE. Our results suggest that both elastin degradation and arterial calcification independently contribute to PAD in PXE.

## 1. Introduction

Pseudoxanthoma elasticum (PXE) is a monogenetic disorder in which mutations in the *ABCC6* gene result in calcification and fragmentation of elastin fibers in the skin, the Bruch’s membrane of the eyes, and the internal elastic lamina of the peripheral arteries [1]. This results clinically in skin loosening and pseudoxanthomatous papules and plaques, severe visual impairment, and peripheral arterial disease [2,3]. The *ABCC6* gene is shown to be involved in the maintenance of systemic levels of inorganic pyrophosphate (PPi) [3]. PPi is a strong inhibitor of calcification, and reduced plasma PPi levels seen in PXE are thought to be the cause of accelerated systemic calcification. Recently, a randomized controlled clinical trial has shown that the bisphosphonate etidronate, a molecular homologue of PPi, can halt arterial calcification progression in PXE [4,5].

Besides accelerated systemic calcification, PXE is characterized by the severe degradation of elastin, the highly elastic component of the extracellular matrix. This might contribute to the pathogenesis of peripheral arterial disease in PXE. Elastin degradation is a prominent feature in lung diseases, including chronic obstructive pulmonary disease (COPD) [6], cystic fibrosis [7], and idiopathic pulmonary fibrosis [8], but it has also been shown to be associated with increased carotid femoral pulse wave velocity [9] and abdominal aortic aneurysms [10]. Elastin degradation and calcification have been shown to interact, and the different processes might amplify each other [11]. Degraded elastin molecules are thought to form a scaffold for calcium ions to bind to and therefore contribute to calcium precipitation. Calcification of elastin molecules, on the other hand, leads to the upregulation of matrix metalloproteinases (MMPs), which may degrade elastin molecules [12].

Since elastin fragmentation is a prominent feature of PXE, results in arterial stiffening, and is hypothesized to accelerate arterial calcification [13], elastin degradation might contribute to peripheral arterial disease in PXE. Plasma and urine levels of desmosines, markers of systemic elastin degradation, were indeed shown to be significantly higher in PXE patients compared to controls [14]. No studies, however, have looked into the association of plasma desmosines with (systemic) arterial calcification and peripheral arterial disease. This study aims to compare plasma desmosines between PXE patients and healthy controls, and to investigate the association between plasma desmosines and (1) arterial calcification, (2) peripheral arterial disease, and (3) peripheral arterial disease independent of arterial calcification in PXE.

## 2. Methods

### 2.1. Participants and Demographics

PXE patients were prospectively recruited from the Dutch National Expertise Center for PXE, located in the University Medical Center in Utrecht, the Netherlands. Healthy controls were recruited from the families and acquaintances of PXE patients. All PXE patients had a proven clinical diagnosis of PXE based on the revised criteria by Plomp et al. (2010) [2]. In short, patients had to have at least two of the following three criteria: skin involvement (pseudoxanthoma), eye involvement (peau d’orange and/or angioid streaks), or genetic confirmation (two pathogenic mutations in the *ABCC6* gene) [2]. Ninety-three PXE patients and seventy-two healthy controls were included in this study. Data on length, weight, blood pressure, low-density lipoprotein (LDL)-cholesterol, and estimated glomerular filtration rate (eGFR) was obtained from all PXE patients and controls. The eGFR was measured since plasma desmosines are cleared by the kidneys. The body mass index was calculated as the weight in kilograms divided by the length in square meters. A medical history on type 2 diabetes mellitus (DM), medication use, and smoking status was obtained from the medical files of all PXE patients. This study was approved by the medical ethical review board of the UMC Utrecht (IRB: 15–522, 16–622, 18–767). All PXE patients and healthy controls gave written informed consent for blood collection, and all PXE patients gave written informed consent for the use of their medical files for research purposes. The data that support the findings of this study are available from the corresponding author upon reasonable request.

### 2.2. Plasma Desmosines

Plasma desmosines were measured in ethylenediaminetetraacetic acid (EDTA) plasma. Both the desmosine and isodesmosine fractions were quantified with liquid chromatography-tandem mass spectrometry (LC-MS/MS) as previously described [15,16]. Total plasma desmosines (desmosine + isodesmosine) were used as a measure for systemic elastin degradation.

### 2.3. Arterial Calcification and Vascular Function Tests

Arterial calcification mass was measured on low-dose (<3 mSv for a 70 kg adult) whole-body computed tomography (CT) scans (>40 detector row scanners, Siemens (Erlangen, Germany) or Philips (Cleveland, OH, USA) reconstructed at slice thickness 5 mm, with 4 mm increment, 100 or 120 kVp) in the carotid siphon, common carotid artery, coronary arteries, thoracic and abdominal aorta, iliac arteries, and the femoral and crural arteries. A peripheral arterial calcification mass score was calculated by summing the mass scores in the femoral and crural arteries. A total arterial calcification mass score was calculated by summing the mass scores in all abovementioned arterial beds. Arterial calcifications were defined as hyperdense arterial wall lesions. Calcification mass scores were computed as the product of the volume of the lesion in mL and the mean attenuation in Hounsfield units (HU). A threshold of 130 HU was used for 120 kVp scans and 145 HU for 100 kVp scans to adjust for differences in scanner settings, since this difference has been shown to affect calcification mass scores [17].

The ankle brachial index (ABI) after treadmill test was used as a measure for peripheral arterial disease. ABI measurements were performed by experienced technicians. To calculate the ABI, the systolic blood pressure was measured in the left and right brachial arteries, the tibial posterior arteries, and in the dorsal pedal arteries. During the treadmill test, patients were encouraged to walk on a treadmill with a 10% slope and a speed of 3.5 km/h for 6 min. Patients who stopped prematurely due to pain (claudication) were encouraged to continue walking as soon as possible. The ABI was measured several times in each leg during recovery in a supine position. The ABI was based on the lowest SBP of the a. tibialis posterior or a. dorsalis pedis. The lowest value of these ABI measurements (i.e., the most diseased leg) was used as the post-treadmill test ABI. Peripheral arterial disease was defined as an ABI < 0.9. Patient-reported claudication was quantified based on the Fontaine classification. Patients were classified as having no PAD (ABI > 0.9), asymptomatic PAD (ABI < 0.9 without claudication), and symptomatic PAD (ABI < 0.9 with claudication).

### 2.4. Pulmonary Abnormalities

Pulmonary abnormalities were scored in all PXE patients on the low-dose CT scans by an experienced thorax radiologist with >10 years of experience. The extent of emphysema was scored on a four-point scale (non-existent, a trace (<1% of total lung volume), mild (1–5% of total lung volume), moderate (>5%)), as previously defined [18]. In addition, the presence of pulmonary fibrosis and bronchiectasis were scored.

### 2.5. Statistical Analysis

Descriptive statistics were presented as mean ± SD for normally distributed continuous variables, median (Q1–Q3) for non-normally distributed variables, or *n* (%) for categorical variables. Data was analyzed with the Student’s *t*-test for normally distributed variables, the Mann–Whitney *U* test for non-normally distributed variables, and the χ^2^ test for categorical variables. The difference in plasma desmosines was tested with the Mann–Whitney *U* test. In addition, to adjust for differences in age and sex, a linear regression model was built with PXE or control as the determinant and plasma desmosines as the outcome, adjusting for age and sex. The correlation between age, arterial calcification mass scores, and ABI and plasma desmosines was tested with the Pearson correlation coefficient. The difference in plasma desmosines between PXE patients with and without PAD and in patients with asymptomatic and symptomatic PAD was tested with the Mann–Whitney *U* test. In addition, to adjust for possible confounders, linear regression models were built with logarithmic transformed arterial calcification mass scores, ABI, peripheral arterial disease or Fontaine classification as the determinants, and plasma desmosines as the outcome. Different models were built adjusting for age and sex, age, sex, BMI, smoking, and DM and age, sex, BMI, smoking, DM and pulmonary abnormalities. In addition, to further test the individual roles of arterial calcification and plasma desmosines in ABI and peripheral arterial disease, regression models were built with plasma desmosines as the determinant and ABI or peripheral arterial disease as the outcome, adjusting for age, sex, BMI, smoking, DM and pulmonary abnormalities, and peripheral or total arterial calcification mass. To assess for interactions, the *p* value of the cross-product of plasma desmosines and peripheral or total arterial calcification mass in the linear (ABI) and logistic (peripheral arterial disease) regression models were used.

All data analyses were performed in SPSS v25. Figures were made in Rstudio v3.5.1. A *p*-value < 0.05 was regarded as statistically significant.

## 3. Results

### 3.1. Baseline Characteristics

**Figure 1:**
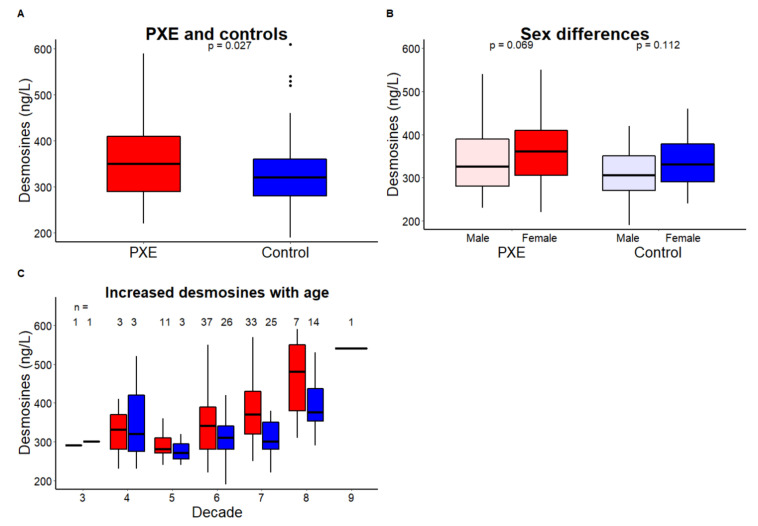
Plasma desmosines in PXE patients and healthy controls. (**A**) PXE patients (red) have significantly higher plasma desmosines than healthy controls (blue); (**B**) Male (light) and female (dark) differences in PXE patients (red) and healthy controls (blue); and (**C**) increased plasma desmosines with age in PXE (red) and healthy controls (blue).

In this study, 93 PXE patients (age range 28–80 years, 51 females) and 72 healthy controls (age range 23–76 years, 30 females) were included between December 2016 and November 2019. PXE patients had lower LDL cholesterol than controls (3.0 ± 1.1 vs. 3.4 ± 1.2 mmol/L, *p* < 0.01), but often used cholesterol lowering medication (67%). Seven PXE patients (8%) had undergone revascularization surgery. PXE patients had higher plasma desmosines (350 (290–410) ng/L vs. 320 (280–360) ng/L, *p* = 0.02, Table 1). This difference remained significant after adjustment for age and sex (β (95%CI): −37 (−64; −9) ng/L, *p* < 0.01). Plasma desmosines were significantly correlated with age in both PXE patients (*R* = 0.47, *p* < 0.01) and controls (*R* = 0.27, *p* = 0.02). Although not statistically significant, females had slightly higher plasma desmosines in both PXE patients (360 (300–410) ng/L vs. 330 (280–390) ng/L, *p* = 0.07) and controls (330 (290–390) ng/L vs. 310 (270–350) ng/L, *p* = 0.11, Figure 1 and Supplementary Figure S1).

### 3.2. Association of Plasma Desmosine with Ankle Brachial Index and Peripheral Arterial Disease

**Figure 2:**
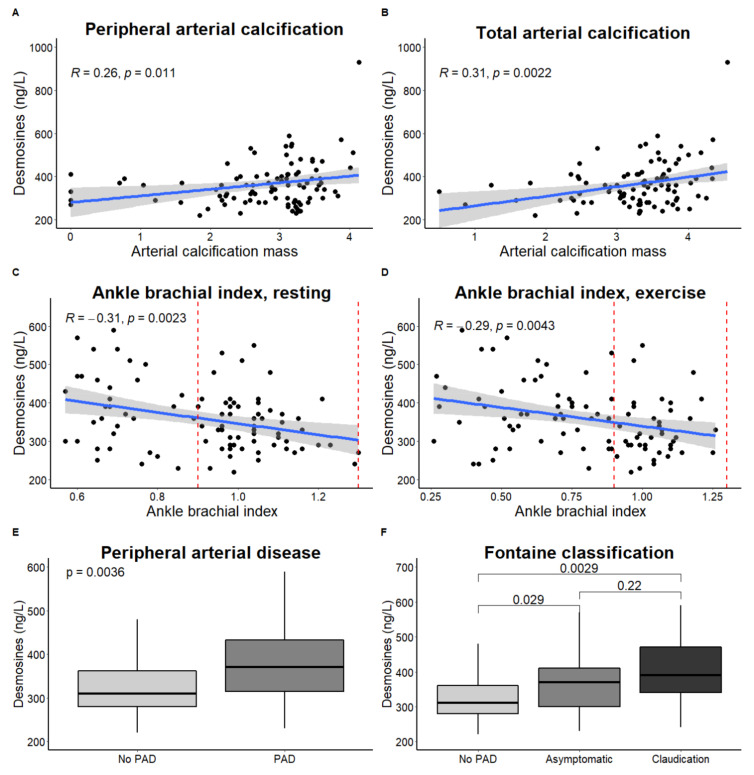
Correlation between plasma desmosines and disease severity in PXE. Plasma desmosines were significantly correlated with peripheral arterial calcification mass (**A**), total arterial calcification mass (**B**), resting and exercise ankle brachial index ((**C**,**D**); red lines indicate normal ABI (0.9–1.3)). PXE patients with peripheral arterial disease had higher plasma desmosines (**E**). PXE patients with asymptomatic and symptomatic PAD had higher plasma desmosines than patients without PAD (**F**). Boxplots show lowest non-outlier, Q1, median, Q3, highest non-outlier.

A significant correlation was found between plasma desmosines and peripheral and total arterial calcification mass as well as ankle brachial index in PXE patients (Figure 2). Patients with peripheral arterial disease had significantly higher levels of plasma desmosines. Both patients with asymptomatic and symptomatic PAD had higher plasma desmosines than patients without PAD. After adjustment for age, sex, body mass index (BMI), smoking, DM, and pulmonary abnormalities, lower ABI was associated with higher plasma desmosine (β (95%CI): −68 (−132; −3) ng/L, *p* = 0.04), and worse ABI is therefore associated with higher elastin degradation. In addition, having peripheral arterial disease and a higher Fontaine classification were associated with higher plasma desmosines (β (95%CI): 40 (7; 73) ng/L, *p* = 0.02 and β (95%CI): 30 (6; 53), *p* = 0.02, respectively), reflecting more elastin degradation (Table 2). Exclusion of patients with pulmonary abnormalities (Table 2) or patients who had undergone revascularization surgery did not significantly affect these results (Supplemental Table S1).

When adjusted for peripheral arterial calcification mass (β (95%CI): −0.71 (−1.38; −0.03) µg/L) or total arterial calcification mass (β (95%CI): −0.71 (−1.39; −0.03 µg/L), plasma desmosines were significantly associated with lower ABI. No significant interaction between plasma desmosines and peripheral or total arterial calcification mass was found (*p* = 0.63 for interaction plasma desmosines and peripheral arterial calcification, *p* = 0.57 for interaction plasma desmosine and total arterial calcification mass) (Table 3), which shows that both elastin degradation and arterial calcification mass independently contribute to lowering ABI in PXE.

## 4. Discussion

Here, we confirm that PXE patients have significantly higher plasma desmosines, as a measure of systemic elastin degradation, compared to healthy controls. This suggests that, besides calcification, enhanced elastin degradation may contribute to the reduced skin elasticity [2] and PAD seen in PXE [19]. Furthermore, we show that plasma desmosines are associated with the severity of peripheral arterial disease independent of the calcification burden in the legs. Both elastin degradation and calcification may therefore be a cause of disease manifestations in PXE.

The association between plasma desmosines and ABI shows that elastin degradation is involved in arterial functioning and contributes to peripheral arterial disease in PXE. These findings are in line with research in patients with COPD that showed that plasma desmosines are correlated with vascular function tests, including pulse wave velocity (ρ = 0.15, *p* < 0.05), and increased in patients with a history of ischaemic heart disease (0.43 ± 0.18 ng/mL) compared to those without (0.37 ± 0.15 ng/mL, *p* = 0.05) [9]. PXE is characterized by increased calcification, and low systemic plasma levels of the calcification inhibitor PPi are thought to be the cause of this increased propensity for calcification [3]. Since increased calcium content is speculated to lead to increased elastase activity and accelerated elastin degradation [11,20], we hypothesized that the high plasma desmosines levels in PXE would be at least partially mediated by calcification. However, we could not confirm in our study that plasma desmosines are associated with arterial calcification mass scores after adjustment for age, sex, BMI, smoking, DM, and pulmonary abnormalities. In addition, both plasma desmosines and arterial calcification were independently associated with ABI, peripheral arterial disease, and with Fontaine classification, which suggests that these different processes independently contribute to the pathogenesis of PXE. Although low ABI is a measure for the degree of stenosis in the vasculature between the aorta and the peripheral arteries in the legs [21], a cross-sectional study showed reduced arterial compliance and aortic distensibility in individuals with low ABI [22]. This suggests that besides stenosis, arterial stiffness also contributes to peripheral arterial disease. Although differing results have been found regarding arterial stiffness in PXE [19,23,24,25,26], we speculate that large arterial calcifications might contribute to arterial stenosis, whereas the increased elastin degradation might reflect subsequent arterial stiffness. These findings would challenge the current theory that low PPi is the sole cause of peripheral arterial disease in PXE, and these findings require further investigation.

Besides atherosclerosis, arterial calcifications in PXE are seen along the internal elastic lamina and in the medial layer of the arterial wall [27]. Such calcifications are known to contribute to elevated ABI and might therefore result in falsely normal ABI values [28]. Based on the resting ABI, 37% of PXE patients had PAD, whereas this increased to 57% after exercise. This emphasizes the clinical benefit of ABI after treadmill test in addition to the resting ABI for the diagnosis of PAD in PXE. Interestingly, only 23% of PXE patients reported claudication. These findings are in line with previous literature on PAD in PXE, where only 56% of PXE patients with an ABI < 0.90 had intermittent claudication [26]. It is speculated that efficient collateral circulation in PXE contributes to this discrepancy between objective and subjective PAD.

Although plasma desmosines were significantly higher in PXE patients than in controls, there is a considerable overlap between the groups. This measurement can therefore not be used to distinguish PXE patients from other populations. Previous research investigating plasma desmosines in PXE patients found much higher values (up to 200 times) in both patients and controls compared to our study [14]. This might be due to the differences in the methods that were used to quantify plasma desmosines. The high-performance liquid chromatography-capillary electrophoresis (HPLC-CE) method used by Annovazzi et al. (2004) [14] is less specific for plasma desmosines than the LC-MS/MS method we used, since it lacks the specificity to differentiate desmosines from other elastin-derived peptides [29]. Indeed, our plasma levels are more in line with other studies reported in the literature [7,8,10,20].

Strengths of this study include the large number of PXE patients, the extensive arterial calcification and vascular function measurements that were performed, and the use of LC-MS/MS for determination of plasma desmosines. A limitation is that we did not perform CT scanning in the healthy controls. Therefore, we could not assess the vascular calcifications and pulmonary abnormalities in the healthy controls. Since pulmonary abnormalities did affect the desmosine levels in PXE, this might have affected our results to some extent. However, since they were relatively mild in the PXE patients and a frequent finding in the general population [30], we think that it did not explain the difference we found between the patients and the controls. In addition, the lack of CT scans and ABI measurements made it impossible to investigate the association between plasma desmosines and arterial calcification mass and peripheral arterial disease in healthy controls. Due to the cross-sectional study design, causality cannot be assessed. It might therefore be that PAD in PXE results in increased elastin degradation. Future research might want to assess urinary desmosines in addition to plasma desmosines in order to provide a more complete picture of the role of elastin degradation in PXE. In addition, longitudinal studies are required to shed more light on the role of elastin degradation in the progression of PXE.

## 5. Conclusions

We showed that plasma desmosines, as a measure of systemic elastin degradation, were increased in PXE patients compared to controls. The negative association between plasma desmosines and ABI emphasizes the role of elastin degradation in peripheral arterial disease in PXE. No association between plasma desmosines and arterial calcification mass was found. Plasma desmosines and arterial calcification mass were independently inversely associated with ABI in PXE patients, which suggests that both elastin degradation and arterial calcification contribute to peripheral arterial disease in PXE.

## Figures and Tables

**Table 1:** Baseline characteristics PXE patients and controls.

Characteristic	PXE (*n* = 93)	Control (*n* = 72)	*p* Value
Age, years	57 ± 9	60 ± 10	0.10
Female sex, *n* (%)	51 (55%)	30 (42%)	0.09
Body mass index	26.1 ± 3.7	25.4 ± 4.1	0.32
Systolic blood pressure (mmHg)	135 ± 18	131 ± 16	0.21
Diastolic blood pressure (mmHg)	77 ± 10	78 ± 10	0.53
Revascularization surgery (PTA or bypass)	7 (8%)		
Blood pressure lowering medication, *n* (%)	27 (29%)		
Lipid lowering medication, *n* (%)	62 (67%)		
Smoking current, *n* (%)	15 (16%)		
Smoking former, *n* (%)	61 (66%)		
Diabetes mellitus 2, *n* (%)	5 (5%)		
LDL cholesterol (mmol/L)	3.0 ± 1.1	3.4 ± 1.2	<0.01 *
Glomerular filtration rate (mL/min/1.73 m²)	90 (80–90)	90 (82–90)	0.82
Desmosines (ng/L)	350 (290–410)	320 (280–360)	0.02 *
**Arterial calcification mass**	**PXE (*n* = 93)**	**-**	
Peripheral arteries, mass score	1190 (262–1892)	-	
Total, mass score	2281 (946–4951)	-	
**Arterial function test**	**PXE (*n* = 92)**	**-**	
Ankle brachial index, resting	0.92 ± 0.19		
Peripheral arterial disease (ABI < 0.9), resting, *n* (%)	34 (37%)		
Ankle brachial index, exercise	0.80 ± 0.26	-	
Peripheral arterial disease (ABI < 0.9), exercise, *n* (%)	52 (57%)	-	
**Fontaine classification**	**PXE (*n* = 93)**		
No PAD	41 (44%)		
Fontaine 1: asymptomatic	31 (33%)		
Fontaine 2: claudication	21 (23%)		
**Pulmonary abnormalities**			
Pulmonary abnormalities, *n* (%)	11 (12%)	-	
Emphysema, *n* (%)	8 (9%)	-	
Trace (<1% of lung volume)	4 (4%)	-	
Mild (1–5% of lung volume)	3 (3%)	-	
Moderate (5–20% of lung volume)	1 (1%)	-	
Pulmonary fibrosis, *n* (%)	2 (2%)	-	
Bronchiectasis, *n* (%)	1 (1%)	-	

Data presented as mean ± SD, median (Q1–Q3), or number (%) when appropriate. Data was analyzed with the Student’s *t*-test for normally distributed variables, the Mann–Whitney *U* test for non-normally distributed variables, and the χ^2^ test for categorical variables. A *p* < 0.05 was regarded as statistically significant. PXE: pseudoxanthoma elasticum; ABI: ankle brachial index; LDL: low-density lipoprotein; PAD: peripheral arterial disease, PTA: percutaneous transluminal angioplasty. * = *p*-value < 0.05.

**Table 2:** Association plasma desmosines and disease severity in pseudoxanthoma elasticum. After adjustment for age, sex, BMI, smoking, DM, and pulmonary abnormalities, lower ABI and having peripheral arterial disease (PAD) are associated with higher plasma desmosines. Worse ABI and PAD are therefore associated with higher elastin degradation.

Total Group (*n =* 93)	Age and Sex Adjusted β (95%CI)	Age, Sex, BMI, Smoking, and DM Adjusted β (95%CI)	Age, Sex, BMI, Smoking, DM, and Pulmonary Abnormalities Adjusted β (95%CI)
Peripheral arterial calcification mass	17 (−9; 43)	15 (−11; 40)	7 (−17; 31)
Total arterial calcification mass	26 (−8; 60)	25 (−9; 59)	12 (−21; 45)
Ankle brachial index	−72 (−132; −12) *	−77 (−140; −13) *	−68 (−132; −3) *
Peripheral arterial disease	42 (10; 73) **	44 (11; 77) **	40 (7; 73) *
Fontaine classification	35 (12; 59) **	41 (17; 64) **	30 (6; 53) *
**Patients with CT and blood collection on same day (*n* = 73)**			
Peripheral arterial calcification mass	57 (10; 104) *	54 (9; 99) *	28 (−16; 72)
Total arterial calcification mass	59 (7; 110) *	55 (4; 105) *	22 (−28; 72)
**Patients with pulmonary abnormalities excluded (*n* = 82)**			
Peripheral arterial calcification mass	3 (−19; 25)	3 (−19; 25)	-
Total arterial calcification mass	−1 (−30; 28)	−0 (−31; 30)	-
Ankle brachial index	−57 (−119; 5)	−69 (−135; −3) *	-
Peripheral arterial disease	34 (2; 67) *	38 (5; 72) *	-
Fontaine classification	25 (5; 46) *	30 (8; 52) **	-

Linear regression models were built with arterial calcification mass scores, ankle brachial index, peripheral arterial disease or Fontaine classification as the determinants and plasma desmosines in ng/L as the outcome. Models were adjusted for age and sex; age sex, BMI, smoking, and DM; and age, sex, BMI, smoking, DM, and pulmonary abnormalities. BMI: body mass index, DM: type 2 diabetes mellitus. * = *p* < 0.05, ** = *p* < 0.01.

**Table 3:** Independent associations of plasma desmosine and arterial calcification with ankle brachial index in PXE.

	Adjusted for Peripheral Arterial Calcification β/OR (95%CI)	*p*-Value for Interaction	Adjusted for Total Arterial Calcification β/OR (95%CI)	*p*-Value for Interaction
Ankle brachial index	−0.71 (−1.38; −0.03) *	0.63	−0.71 (−1.39; −0.03) *	0.57
Peripheral arterial disease	1.01 (1.00; 1.02) *	0.09	1.01 (1.00; 1.02) *	0.65
Fontaine classification	1.01 (1.00; 1.01) *	0.07	1.01 (1.00; 1.01) *	0.38

Linear regression models were built with plasma desmosines (in µg/L) as the determinant and ABI as the outcome, logistic regression models were built with plasma desmosine (in ng/L) as the determinant and presence of peripheral arterial disease as the outcome, and ordinal logistic regression models were built with plasma desmosine (in ng/L) as the determinant and Fontaine classification as the outcome. Models were adjusted for age, sex, BMI, smoking, DM, and pulmonary abnormalities and peripheral or total arterial calcification mass. To assess the interaction between plasma desmosines and arterial calcification mass, the *p* value of the cross-product of plasma desmosines and peripheral and total arterial calcification mass in the linear regression model was reported. OR: odds ratio. * = *p* value < 0.05.

## Supplementary Materials

The following are available online at https://www.mdpi.com/2077-0383/9/9/2771/s1, Figure S1: Desmosine in PXE including outliers, Table S1: Association plasma desmosines and disease severity in pseudoxanthoma elasticum with patients with revascularization surgery excluded.
