# Supplementary material for: Increased Elastin Degradation in Pseudoxanthoma Elasticum Is Associated with Peripheral Arterial Disease Independent of Calcification
Source: J Clin Med. 2020 Aug 26;9(9):2771. doi: 10.3390/jcm9092771 (PMC7563692; doi:10.3390/jcm9092771)
Supplement: Supplementary file 1 [file jcm-09-02771-s001.pdf]

Supplemental figure 1: Desmosine in PXE including outliers. A) difference in plasma desmosines between PXE patients and healthy controls, B) difference in plasma desmosines levels between male and female PXE patients and controls, C) difference in plasma desmosines in PXE patients with and without peripheral arterial disease and D) difference in plasma desmosines in PXE patients without, with asymptomatic and with symptomatic PAD.

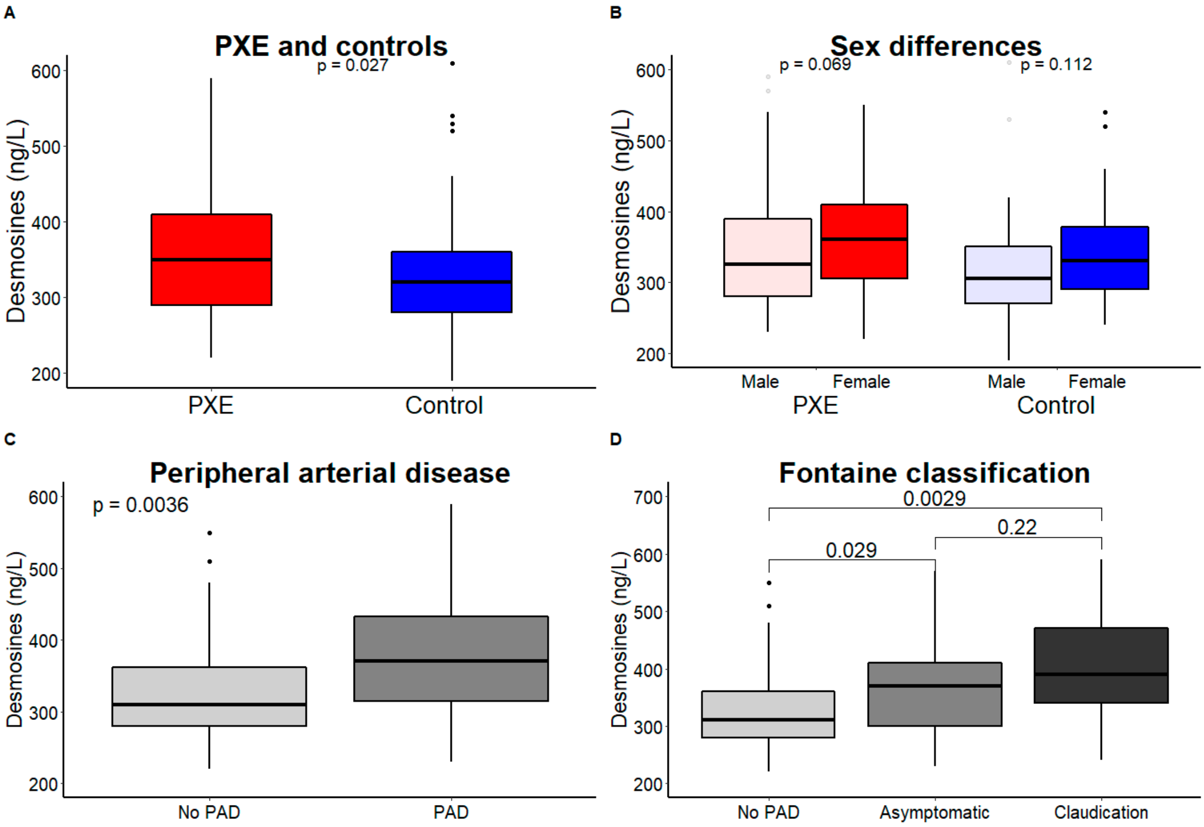

Supplemental table 1. Association plasma desmosines and disease severity in pseudoxanthoma elasticum with patients with revascularization surgery excluded. After adjustment for age, sex and pulmonary abnormalities, lower ABI and having peripheral arterial disease (PAD) are associated with higher plasma desmosine. Worse ABI and PAD are therefore associated with higher elastin degradation.

Total group (n=86)	**Age and sex adjusted β [95%CI]**	**Age, sex, BMI, smoking and DM adjusted β [95%CI]**	**Age, sex, BMI, smoking, DM and pulmonary abnormalities adjusted β [95%CI]**
Peripheral arterial calcification mass	3 [-22;27]	2 [-24;27]	-1 [-26;24]
Total arterial calcification mass	4 [-27;36]	2 [-31;36]	-1 [-35;32]
Ankle brachial index	-74 [-137;-10]*	-79 [-146;-11]*	-69 [-137;-0]*
Peripheral arterial disease	44 [11;76]*	46 [12;80]**	41 [7;76]
Fontaine classification	30 [9;52]**	31 [8;54]**	27 [4;51]
Patients with CT and blood collection on same day (n=69)			
Peripheral arterial calcification mass	21 [-20;62]	21 [-21;64]	16 [-27;58]
Total arterial calcification mass	19 [-26;65]	18 [-29;66]	9 [-39;58]
Patients with pulmonary abnormalities excluded (n = 76)			
Peripheral arterial calcification mass	-1 [-25;24]	-0 [-25;25]	
Total arterial calcification mass	-7 [-38;25]	-6 [-40;26]	
Ankle brachial index	-58 [-124;8]	-70 [-141;-0]*	
Peripheral arterial disease	36 [2;70]*	40 [4;75]*	
Fontaine classification	28 [5;51]*	30 [7;54]*	

Linear regression models were built with arterial calcification mass scores, ankle brachial index, peripheral arterial disease or Fontaine classification as the determinants and plasma desmosine in ng/L as the outcome. Models were adjusted for age and sex and age, sex and pulmonary abnormalities. * = p<0.05, ** = p<0.01.
